# Efficacy and safety of albendazole alone *versus* albendazole in combination with ivermectin for the treatment of *Trichuris trichiura* infections: An open-label, randomized controlled superiority trial in south-western Uganda

**DOI:** 10.1371/journal.pntd.0012687

**Published:** 2024-11-26

**Authors:** Marta Sólveig Palmeirim, Eveline Hürlimann, Prudence Beinamaryo, Hilda Kyarisiima, Betty Nabatte, Jan Hattendorf, Peter Steinmann, Jennifer Keiser

**Affiliations:** 1 University of Basel, Basel, Switzerland; 2 Swiss Tropical and Public Health Institute, Allschwil, Switzerland; 3 Ministry of Health, Department of Environmental Health, Division of Vector-borne and Neglected Tropical Diseases, Kampala, Uganda; Uniformed Services University: Uniformed Services University of the Health Sciences, UNITED STATES OF AMERICA

## Abstract

*Trichuris trichiura*, a soil-transmitted helminth (STH), often persists after a single dose of anthelminthic treatment. To overcome limited efficacy against *T*. *trichiura* of benzimidazoles (albendazole or mebendazole), the primary drugs used in mass drug administration (MDA) campaigns, the World Health Organization endorses the use of a combination of ivermectin and albendazole as a more effective treatment to be used for preventive chemotherapy. Given observed considerable differences in efficacy of the combination therapy over albendazole monotherapy between different settings, it is necessary to evaluate the performance of the combination before introducing it on a larger scale. This open-label, randomized controlled superiority trial in two Ugandan primary schools enrolled eligible 6- to 12-year-olds positive for *T*. *trichiura*. Participants were randomized 1:1 to receive either a single dose of albendazole alone or co-administered albendazole and ivermectin. Adverse events were monitored at three and 24h post-treatment. Follow-up samples were collected 14 to 21 days post-treatment for efficacy assessment. The combination of albendazole with ivermectin showed superior efficacy against *T*. *trichiura* compared to albendazole alone, both in terms of cure rates (31.3% versus 12.3%, difference 18.9%-points, 95% CI 6.2–31.2, p < 0.004) and in terms of egg reduction rates (ERRs; 91.4% versus 52.7%). A higher cure rate against co-infecting *Ascaris lumbricoides* was observed in the combination compared to the albendazole monotherapy arm (100% versus 83.9%). Both therapies showed an excellent safety profile with few and only mild and transient treatment emergent adverse events observed in the albendazole monotherapy and albendazole plus ivermectin arm (total of 22 and 19 events, respectively). In conclusion, the efficacy of the combination therapy against *T*. *trichiura* in Uganda is superior to that of albendazole alone. Given the high ERRs observed, albendazole-ivermectin might aid in eliminating morbidity, an important target of STH control programs.

**Trial registration** (clinicaltrials.gov): NCT06037876.

## Author summary

Despite several decades of preventive chemotherapy with single-dose albendazole distribution to at-risk populations, soil-transmitted helminth infections remain of public health concern in Uganda. The Ministry of Health has stated interest in the introduction of potentially more efficacious treatment strategies against *T*. *trichiura* such as the use of a combination of two deworming drugs (ivermectin and albendazole) instead of only albendazole as currently used. Given significant differences in efficacy between the combination therapy compared to albendazole monotherapy against *T*. *trichiura* in different settings, we conducted a randomized clinical trial in two Ugandan primary schools to assess whether in Uganda the combination is more efficacious than albendazole alone. The findings of our study confirmed higher efficacy against *T*. *trichiura* in the study area. Both treatment regimens were well tolerated. In conclusion, we conceive that the introduction of the combination therapy is advantageous and its introduction into the existing STH control programme should now be evaluated from a recipient and provider perspective.

## Introduction

Infections with soil-transmitted helminths (STH), namely *Ascaris lumbricoides*, *Trichuris trichiura*, and hookworm species (*Ancylostoma duodenale* and *Necator americanus*) still pose a significant health challenge globally [1]. Presently, nearly 1.5 billion individuals worldwide, predominantly in tropical and subtropical regions, suffer from these parasitic infections [2]. This results in an annual burden of approximately 1.9 million disability-adjusted life years [3]. The consequences of STH infections encompass nutritional deficiencies, anaemia, impaired physical and cognitive development in children, and diminished work performance in adulthood [4,5]. Current control strategies predominantly rely on preventive chemotherapy, involving the routine administration of mebendazole or albendazole to populations at risk. Nevertheless, the currently employed drugs have been shown to have low efficacy against infections with *T*. *trichiura* [6,7].

In 2017, WHO added the combination of albendazole and ivermectin to their List of Essential Medicines, as it had been shown in several clinical trials and a systematic review to be more efficacious than albendazole alone against STH [7,8]. This shift from a single tablet of albendazole to the combination involves extra costs as ivermectin is not donated. However, since recent years ivermectin is available from generic producers at an affordable cost. Moreover, ivermectin is a weight dependent drug and, therefore, this combination therapy requires individually assessing the number of ivermectin tablets to administer. In the context of mass drug administration where millions of people are treated yearly, this may require a considerable amount of extra resources and time.

The “Feasibility And Cost-Effectiveness of Improved Treatment against helminthiases in children” (FACE IT) project aims at exploring the most feasible and effective approach to the mass administration of the ivermectin-albendazole combination for STH. Prior to the wide implementation of the combination of albendazole and ivermectin, the first step of this project involved implementing a clinical trial to confirm, for the first time, that in Uganda the combination is indeed more efficacious than albendazole alone. The context for this is that a recent multi-country trial found that whereas in Pemba Island (Tanzania) and Lao PDR the combination was significantly more efficacious than albendazole alone, this was not the case in Côte d’Ivoire where both drugs were equally and insufficiently efficacious. One of the reasons behind this might be the existence of a *Trichuris* species phylogenetically distinct from *T*. *trichiura* (*T*. *incognita*) that is less responsive to ivermectin-albendazole [9].

In the current study, we aimed at confirming superiority of the combination of albendazole and ivermectin against *T*. *trichiura* infections compared to albendazole alone in Uganda.

## Methods

### Ethics statement

Ethical approval was obtained from the Uganda National Council for Science and Technology (UNCST, no. HS3160ES), the Vector Control Division Research and Ethics Review Committee (VCDREC, no. VCDR-2023-29) and from the Ethics Committee of North-western and Central Switzerland (EKNZ, no. AO_2023–00066). Written consent was obtained from caregivers of all children participating in this study.

### Trial design

This parallel open-label randomized controlled superiority trial took place in the primary schools of Kahungye, Kabale district, and Rwanzu, Kisoro district, both in south-western Uganda. Participants were allocated 1:1 to receive either a single dose of albendazole alone or albendazole plus ivermectin.

### Randomization and masking

Randomization was performed through a computer-generated sequence with varying block sizes of four and six, stratified into two levels by baseline *T*. *trichiura*-infection intensities (light: 1–999 eggs per gram (EPG), and moderate plus heavy: ≥ 1000 EPG). Treatment allocation was concealed using sealed opaque envelopes, each labelled with a treatment identification code. The person delivering the drugs was not blinded, but outcome-assessing laboratory personnel were.

### Interventions

Eligible schoolchildren were administered a single dose of either albendazole alone or in combination with ivermectin. The number of ivermectin tablets was based on the standard dose pole category each child fell into: one tablet if the child was between 90–119 cm of height; two if between 120–140 cm; three if between 141–158 cm; four if above 158 cm. The dose pole was used as a proxy for weight-based dosing and corresponded to the WHO recommended tools to administer 200 μg/kg of ivermectin if 3 mg tablets are used [10].

### Procedures

Participants’ name, age, sex, and school grade were recorded. Caregivers of children falling within the 6 to 12 years age group attending the study schools were invited to information sessions. During these sessions, a study representative explained the study’s objectives, procedures, benefits, and potential associated risks. Caregivers were given the opportunity to ask questions, and those opting for their child’s participation were asked to provide written informed consent. Caregivers who were unable to read provided a thumbprint, and an impartial witness signed, confirming that the team delivered a comprehensive explanation of all relevant information contained in the informed consent form.

Children with caregiver consent were asked to provide two baseline stool samples to assess infection with *T*. *trichiura*. All children who had at least two of the four Kato-Katz slides positive for *T*. *trichiura* were invited for treatment. Prior to treatment, all invited children who were present underwent clinical and physical examinations.

Consented children received an empty container marked with their unique identification number (ID) for collection of the first stool sample. The subsequent day, they received a second empty container for the second stool sample. The majority of samples were self-collected by participants at schools and were, therefore, fresh. Whenever feasible, the two stool samples were collected on consecutive days. The field laboratory was set up at the study schools themselves, where experienced technicians produced duplicate Kato-Katz thick smears from each sample. Under a light microscope, eggs of *T*. *trichiura* and *A*. *lumbricoides* were counted and hookworm egg presence documented separately within one hour after preparing the Kato-Katz slides to prevent the clearing of hookworm eggs. Quality control for *A*. *lumbricoides* and *T*. *trichiura* was implemented by randomly selecting 10% of the Kato-Katz slides, eliminating their original ID, and relabelling them with a new quality control ID prior to having technicians re-read the selected slides. For hookworm quality control, a portion of each sample was transferred to a new container, and labelled with a new quality control ID to prevent technicians from identifying the sample; technicians were asked to prepare one Kato-Katz slide from each of these containers and read it.

Children who were infected with *T*. *trichiura* in at least two out of four Kato-Katz slides were invited for physical and clinical examinations prior to treatment. Exclusion criteria comprised presence or signs of major systemic illnesses, anthelmintic drug intake within the past four weeks, participation in another experimental research study, and/or known allergy to either albendazole or ivermectin. Weight, height, and dose pole categories based on height were recorded in individual case report forms. Additionally, the children’s temperature was also measured and documented; children who had a fever (≥ 38°C) underwent a malaria rapid diagnostic test. A positive result would lead to exclusion from the randomization and treatment. Finally, a physician assessed their health to ensure eligibility.

Enrolment and drug administration were overseen by the study team. Children were assigned to receive either albendazole alone (400 mg tablet) or the combination of albendazole (400 mg) and ivermectin (200 μg/kg approximated by dose pole height category). In Kahungye primary school, all children had received food prior to treatment, whereas in the Rwanzu primary school they received food one hour post-treatment. Physicians and nurses actively queried each participant for adverse events using a questionnaire at three and 24 hours post-treatment.

During follow-up, conducted between 14 and 21 days after treatment, each participant was requested to provide two additional stool samples. Follow-up samples were also collected at school, if possible on two consecutive days, and underwent the same procedures as described for baseline samples. Participants still found infected with any STH at follow-up were treated with the combination of albendazole (400 mg) and ivermectin (200 μg/kg).

### Endpoints

The primary endpoint of this trial was *T*. *trichiura* infection status of participants 14–21 days post-treatment assessed by duplicate Kato-Katz thick smears. Secondary endpoints were *T*. *trichiura* intensity of infection (egg reduction rate (ERR)) 14–21 days post-treatment also assessed by duplicate Kato-Katz thick smears, and tolerability of treatment (adverse events (AEs) and treatment emergent adverse events (TEAEs)) at three and 24 hours post-treatment.

### Sample size

Based on data from a randomized controlled trial conducted in Côte d’Ivoire and Tanzania [11], we predicted a true CR of 8% for albendazole monotherapy and 30% for the combination of albendazole and ivermectin against *T*. *trichiura* in children aged 6–12 years. Hence, enrolling 64 participants per arm was estimated to be sufficient to identify a statistically significant difference with 90% power using a two-sided 5% significance level. To account for a loss to follow-up of 15%, we planned to recruit 75 schoolchildren per treatment arm, resulting in a total of 150 participants.

### Statistical methods

The primary analysis of trial data was performed according to the intention-to-treat principles, using the available case population, which included all participants with any primary endpoint data (i.e., at least one stool sample result at follow-up). Following this, a per-protocol analysis was conducted. Cure rates were computed as the percentage of participants positive for eggs at baseline who transitioned to egg-negative status post-treatment. Differences among CRs were assessed using a melded binomial test with mid-p correction, and the analysis was supplemented by adjusted logistic regressions using the stratification variable as additional predictor.

Egg counts per gram were determined by calculating the mean egg counts from quadruplicate Kato-Katz thick smears and multiplying the result by a factor of 24. Egg reduction rates, based on the geometric mean (GM) and arithmetic mean (AM) egg counts for each treatment arm before and after treatment, were calculated using the following formulas (Eqs 1 and 2):

$${ERR}_{GM}=1-\frac{e^{\frac{1}{n}\sum \log\left({EPG}_{follow-up}+1\right)}-1}{e^{\frac{1}{n}\sum \log\left({EPG}_{baseline}+1\right)}-1}$$
**Eq 1.** Geometric mean egg reduction rate.

$${ERR}_{AM}=1-\frac{\frac{1}{n}\sum {EPG}_{follow-up}}{\frac{1}{n}\sum {EPG}_{baseline}}$$
**Eq 2.** Arithmetic mean egg reduction rate.

The Bootstrap resampling method with 5,000 replicates was used to estimate 95% confidence intervals (CIs) for geometric ERRs and the difference between the ERRs.

Safety outcomes were not assessed through statistical testing and adverse events are presented in frequency tables. Data were analyzed using R software version 4.0.3 and Stata 18. Specific symptoms observed post-treatment were considered TEAEs if they were not already present before treatment or if they worsened compared to pre-treatment severity.

## Results

### Trial participation

**Fig 1:**
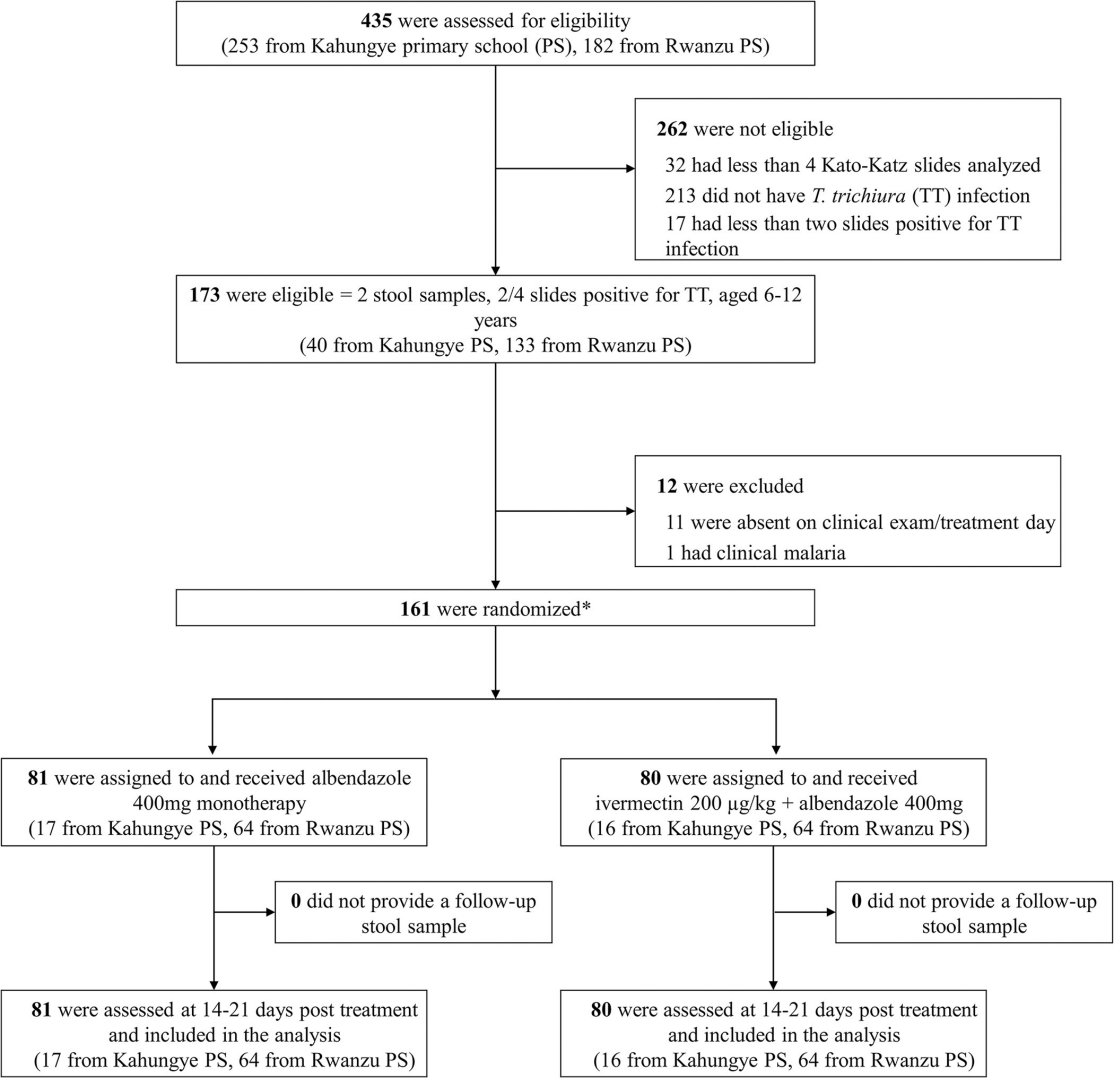
Participant flow diagram. PS, primary school; TT, *Trichuris trichiura*. *Including one participant with <4 Kato-Katz slides analysed, but 2 slides positive for TT that was erroneously randomized.

Conducted from October 9, 2023, to November 14, 2023, this open-label clinical trial involved the screening of 435 children for *T*. *trichiura* infections in two primary schools, Rwanzu and Kahungye, situated in the southwestern regions of Uganda. Among the screened participants, 402 provided two stool samples, and of these, 189 tested positive for *T*. *trichiura*. A total of 91% (173/189) met the minimum parasitological eligibility criteria (at least two out of four Kato-Katz slides positive). Eleven children were absent on treatment day, and one was excluded because he/she had a fever and tested positive for malaria. Consequently, 161 children were successfully randomized, including one child erroneously included who had two slides positive but had only provided one stool sample (Fig 1). Of the trial cohort children 33 belonged to Kahungye and 128 to Rwanzu primary school. Among all participants, 81 were included in the albendazole alone treatment arm and 80 in the combination treatment arm. No child was lost to follow-up, meaning all randomized children provided follow-up stool samples, although 9 children provided only one follow-up sample.

Of the 435 children who were screened, 46.0% were infected with *T*. *trichiura*, 67.4% with *A*. *lumbricoides* and 3.7% with hookworm (S1 Table). For *T*. *trichiura*, 90% of infections were light and 10% were moderate. In the case of *A*. *lumbricoides*, more than half of infections were either moderate (52.6%) or heavy (2.4%).

### Baseline characteristics of trial cohort

Baseline characteristics demonstrated comparability between participants in both treatment arms regarding age, sex, weight, height, and *T*. *trichiura* baseline infection intensity (Table 1). In both treatment arms about three quarters of the children (76.5% and 73.8% in the albendazole alone and albendazole plus ivermectin arm, respectively) were co-infected with *A*. *lumbricoides*, whereof more than half of the infections were of moderate intensity and in the ivermectin-albendazole arm four (6.8%) children had heavy intensity infections. Hookworm infections were rare with two and one individuals infected in the albendazole and the ivermectin-albendazole arm, respectively.

**Table 1:** Baseline characteristics of all randomized children stratified by treatment arm.

	Albendazole alone N = 81	Albendazole plus ivermectin N = 80
Mean age [years]	9.4 (2.2)	9.6 (1.9)
Girls	47 (58.0%)	43 (53.8%)
Mean weight [kg]	25.5 (6.0)	25.8 (5.7)
Mean height [cm]	127.1 (11.8)	126.7 (15.5)
***Trichuris trichiura***		
EPG median	108 (36–384)	237 (51–540)
EPG geometric mean	133.0	180.7
EPG arithmetic mean	404.2	507.8
Infection intensity		
Light (1–999 EPG)	71 (87.7%)	72 (90.0%)
Moderate (1000–9999 EPG)	10 (12.3%)	8 (10.0%)
Heavy (≥10000 EPG)	0 (0.0%)	0 (0.0%)
***Ascaris lumbricoides***		
Infected children	62 (76.5%)	59 (73.8%)
EPG median	5955 (1248–13068)	7908 (3540–17460)
EPG geometric mean	3857.8	6202.7
EPG arithmetic mean	9634.4	14847.1
Infection intensity		
Light (1–4999 EPG)	27 (43.6%)	23 (39.0%)
Moderate (5,000–49999 EPG)	35 (56.4%)	32 (54.2)
Heavy (≥50000 EPG)	0 (0.0%)	4 (6.8%)
**Hookworm**		
Infected children	2 (2.5%)	1 (1.3%)

Data are mean (SD), median (IQR) or N (%). EPG, eggs per gram of stool.

### Efficacy against *Trichuris trichiura* and co-infecting STH species

The mean ivermectin dose and standard deviation among the 80 children in the combination therapy arm corresponded to 223 ± 51 μg/kg if weight was taken into account. A significant difference in the CRs for *T*. *trichiura* infection was observed between the group treated with albendazole alone (12.3%) and the combination of albendazole plus ivermectin (31.3%; difference 18.9%-points, 95% CI 6.2–31.2, p = 0.004; Table 2). Similarly, a statistical difference was observed between both groups with regard to ERR, with 52.7% in the albendazole-alone arm and a notably higher rate of 91.4% in the combination arm (difference 38.7%-points, 95% CI 23.5–57.8). Adjusting for baseline infection intensity did not alter the point or interval estimate significantly. For co-infections, we observed a higher CR for *A*. *lumbricoides* in the combination arm, in which all 59 baseline infected individuals were cured (100%), compared with 52 out of 62 (83.9%) in the albendazole monotherapy arm. No hookworm infections were observed at follow-up.

**Table 2:** Cure rates (CRs) and egg reduction rates (ERRs) against *Trichuris trichiura*, *Ascaris lumbricoides* and hookworm 14 to 21 days after treatment. Data are n (%) and mean (SD). CR, cure rate; CI, confidence interval; EPG, eggs per gram of stool; ERR, egg reduction rate.

	Albendazole alone	Albendazole plus ivermectin	Difference (95% CI)
***Trichuris trichiura***			
Children positive before treatment	81	80	
Children cured after treatment	10	25	
CR in % (95% CI)	12.3 (6.1–21.5)	31.3 (21.3–42.6)	18.9 (6.2–31.2)
Children with light infections cured	10/71 (14.1%)	23/72 (31.9%)	
Children with moderate infections cured	0/10 (0.0%)	2/8 (25.0%)	
EPG geometric mean			
Before treatment	133.0	180.7	
After treatment	62.9	15.5	
ERR in % (95% CI)	52.7 (33.8–66.8)	91.4 (86.0–95.0)	38.7 (23.5–57.8)
EPG arithmetic mean			
Before treatment	404.2	507.8	
After treatment	284.4	108.0	
ERR in % (95% CI)	29.6 (12.4–45.7)	78.7 (68.8–86.5)	
***Ascaris lumbricoides***			
Children positive before treatment	62	59	
Children cured after treatment	52	59	
CR in % (95% CI)	83.9 (72.3–92.0)	100.0 (93.9–100.0)	16.1 (7.3–26.3)
Children with light infections cured	22/27 (81.5%)	23/23 (100.0%)	
Children with moderate to heavy infections cured	30/35 (85.7%)	36/36 (100%)	
EPG geometric mean			
Before treatment	3857.8	6202.7	
After treatment	1.4	0.0	
ERR (95% CI)	99.96 (99.90–99.99)	100.0 (100.0–100.0)	
EPG arithmetic mean			
Before treatment	9634.4	14847.1	
After treatment	241.6	0.0	
ERR (95% CI)	97.6 (94.7–99.5)	100.0 (100.0–100.0)	
**Hookworm**			
Children positive before treatment	2	1	
Children cured after treatment	2	1	
CR (95% CI)	100.0 (15.8–100.0)	100.0 (2.5–100.0)	

### Tolerability of the treatment regimens

All 161 trial participants were assessed for baseline symptoms and were reassessed for any adverse events (AEs) and treatment emergent adverse events (TEAEs; symptoms not already observed in the participant prior drug administration) three hours post-treatment (Table 3). In total, 159 participants (80 and 79 in the albendazole alone and albendazole plus ivermectin arms, respectively) were reassessed for any AEs and TEAEs 24 hours post-treatment. Prior to drug administration, over half of the participants in either treatment arm (64.2% and 62.5% in the albendazole alone and albendazole plus ivermectin arm, respectively) reported symptoms. Fifteen (18.5%) and 11 (13.8%) participants of the albendazole alone arm and the albendazole plus ivermectin arm, respectively, showed at least one TEAE three hours after treatment. Twenty-four hours post-treatment five (6.3%) and eight (10.1%) participants in the albendazole alone and albendazole plus ivermectin arms, respectively, showed at least one TEAE. All observed TEAEs were of mild grading and no medical intervention was needed at any time point.

**Table 3:** Total numbers of baseline symptoms, adverse events (AE) and treatment emergent adverse events (TEAE), by assessment time-point and treatment arm.

	Before treatment	3h post-treatment	24h post- treatment
**Albendazole alone**			
Total participants assessed*	81	81	80
Participants with symptoms/adverse events (AEs), N (%)	52 (64.2)	53 (65.4)	21 (26.3)
Total no. of AEs	61	60	22
No. of mild AEs, N (%)	61 (100.0)	60 (100.0)	22 (100.0)
No. of moderate AEs, N (%)	0 (0.0)	0 (0.0)	0 (0.0)
Participants with treatment emergent adverse events (TEAEs), N (%)	NA	15 (18.5)	5 (6.3)
No. of TEAEs among AEs, n/N (%)	NA	17/60 (28.3)	5/22 (22.7)
**Albendazole plus ivermectin**			
Total participants assessed*	80	80	79
Participants with symptoms/AEs, N (%)	50 (62.5)	41 (51.3)	23 (29.1)
Total no. of AEs	59	46	28
No. of mild AEs, N (%)	59 (100.0)	46 (100.0)	28 (100.0)
No. of moderate AEs, N (%)	0 (0.0)	0 (0.0)	0 (0.0)
Participants with TEAEs, N (%)	NA	11 (13.8)	8 (10.1)
No. of TEAEs among AEs, n/N (%)	NA	11/46 (23.9)	8/28 (28.6)
**Total**			
Total participants assessed*	161	161	159
Participants with symptoms/AEs, N (%)	102 (63.4)	94 (58.4)	44 (27.7)
Total no. of AEs	120	106	50
No. of mild AEs, N (%)	120 (100.0)	106 (100.0)	50 (100.0)
No. of moderate AEs, N (%)	0 (0.0)	0 (0.0)	0 (0.0)
Participants with TEAEs, N (%)	NA	26 (16.2)	13 (8.2)
No. of TEAEs among AEs, n/N (%)	NA	28/106 (26.4)	13/50 (26.0)

*Two individuals do not have 3h temperature measured (one per arm), two individuals with 24h safety data do not have 24h temperature measured (one per arm)

**Fig 2:**
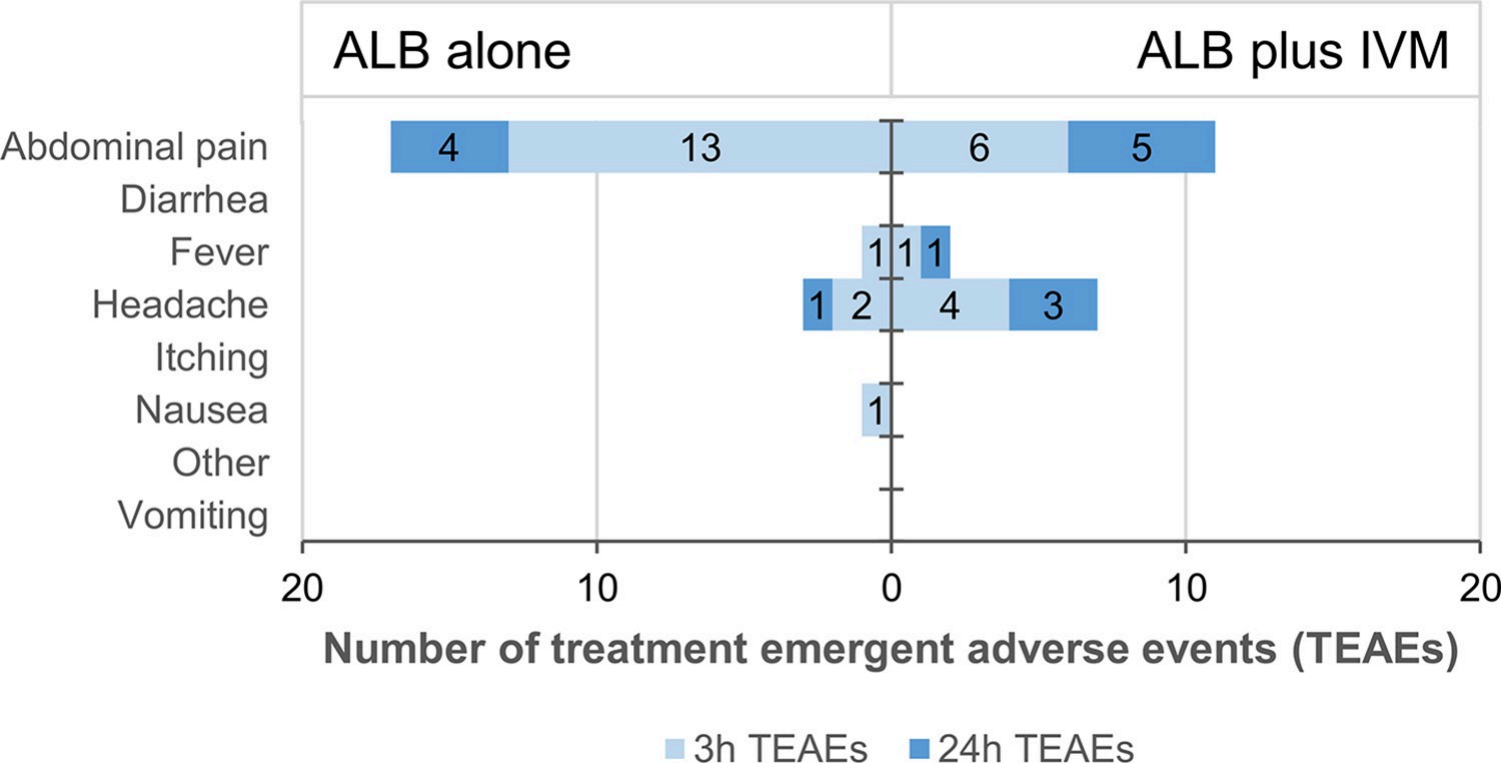
Queried and observed treatment emergent adverse events (TEAEs) 3 and 24 hours after single-dose albendazole or albendazole plus ivermectin combination administration.

The most common TEAEs were abdominal pain in 19 of 161 (11.8%) at three hours and in 9 of 159 (5.7%) participants at 24h post-treatment, and headache in 6 of 161 (3.7%) at three hours and in 4 of 159 (2.5%) participants 24 hours post-treatment (Fig 2).

## Discussion

Current mass drug administration drugs (albendazole or mebendazole) are not sufficiently efficacious to enable us to achieve the WHO’s 2030 goal of reducing the prevalence of moderate and heavy STH infections to below 2% [12]. *T. trichiura* infections are especially challenging to treat [6,13], highlighting the need for alternative treatments to prevent drug resistance and meet global targets. While the combination of ivermectin and albendazole proved more efficacious than albendazole alone in various contexts, recent trials in Côte d’Ivoire contradicted this, revealing no superior performance of the combination over albendazole alone [11,14].

In the current clinical trial, we confirmed that in Uganda the combination treatment is more efficacious against *T*. *trichiura* than albendazole monotherapy. The CR achieved using the combination therapy was over twice as high as the one with albendazole alone. However, the CRs remained below expectations of findings from other settings in eastern Africa and Asia where CRs above 50% were found for the combination therapy [7,11]. Molecular analyses of *T*. *trichiura* eggs and adult worms from Côte d’Ivoire suggest that the non-responsiveness to ivermectin-albendazole may partly relate to genetic variability of the local *Trichuris* species, which has recently been identified as a phylogenetically distinct species, nomenclated *T*. *incognita* [9,15]. Stool samples containing *Trichuris* eggs from this clinical trial have been subjected to genetic characterization and revealed the presence of the *T*. *incognita* cluster in Uganda as well but in lower rates compared to Côte d’Ivoire (14.5% compared to 45.8% of analysed human-infecting *Trichuris* species) [16].

Interestingly, we were also able to demonstrate a benefit of the combination therapy against *A*. *lumbricoides* infections. At follow-up, all children receiving ivermectin-albendazole were no longer shedding eggs in their faeces while ten children in the albendazole only arm still did. This is to some extent unexpected, as in previous clinical trials albendazole was usually effective in clearing *A*. *lumbricoides* infections [11,17]. Yet, the findings are in line with recent studies that also observed reduced efficacy for this parasite in neighbouring areas, such as Rwanda and Ethiopia [18,19]. The results for hookworm in this trial are inconclusive due to the limited number of hookworm-infected children included. However, a recent meta-analysis concluded that the combination therapy offered neither a significant advantage nor disadvantage in efficacy compared to monotherapy for treating hookworm infections [7].

All children tolerated both treatment regimens well and all TEAEs were of transient nature and mild severity. These findings align with those of a literature review and meta-analysis [7]. This is a crucial outcome and encouraging news that supports transitioning from monotherapy to the combination.

The strengths of our study include a robust sample size, complete retention with no loss to follow-up, and its implementation in a region of Uganda that is highly representative of the contexts where this combination therapy could have the greatest impact, given the high prevalence of STH [20]. Limitations of our trial include it not having been double-blinded, and having a very low number of hookworm cases, which prevents us from drawing conclusions about the efficacy of either treatment arm for this particular parasite. In addition to its superior efficacy against *T*. *trichiura* and *A*. *lumbricoides* and its equivalent safety profile, we believe the ivermectin-albendazole combination will offer several other significant advantages over albendazole monotherapy. First, it may help delay the development of drug resistance. Second, by more effectively reducing the worm burden in individuals, the combination therapy could lower transmission rates of these parasites [4]. Finally, the use of ivermectin in addition to albendazole also targets other neglected tropical diseases, such as onchocerciasis, lymphatic filariasis, strongyloidiasis and scabies [21,22].

In conclusion, we believe a shift from preventive chemotherapy using albendazole alone to using the combination treatment in Uganda is justified, particularly in districts where *T*. *trichiura* and *A*. *lumbricoides* infections dominate [20]. The FACE-IT project’s next step will be to explore how to best integrate ivermectin-albendazole co-administration in the existing school-based deworming programme. This will include assessing acceptability among the receiving populations and implementation actors, as well as to document processes and performance of combination treatment distribution compared to routine albendazole mass drug administration.

## Supporting information

S1 CONSORT Checklist CONSORT 2010 checklist of information to include when reporting a randomised trial. (DOC)

S1 Table Proportion of screened children infected with each soil-transmitted helminth (STH) and the respective infection intensities. (DOCX)

S1 Raw Data Anonymized study dataset. (XLSX)
