# Supplementary material for: Efficacy and safety of albendazole alone versus albendazole in combination with ivermectin for the treatment of Trichuris trichiura infections: An open-label, randomized controlled superiority trial in south-western Uganda
Source: PLoS Negl Trop Dis. 2024 Nov 26;18(11):e0012687. doi: 10.1371/journal.pntd.0012687 (PMC11630569; doi:10.1371/journal.pntd.0012687)
Supplement: S1 Table — (DOCX) [file pntd.0012687.s002.docx]

**S1 Table.** Proportion of screened children infected with each soil-transmitted helminth (STH) and the respective infection intensities.

|  |  | Kahungye | Rwanzu | Total |
| --- | --- | --- | --- | --- |
| No. examined* | | 253 (58.2) | 182 (41.8) | 435 (100.0) |
| Infected with *Trichuris trichiura* | | 56 (22.1) | 144 (79.1) | 200 (46.0) |
|  | Light-intensity infection | 56 (100.0) | 124 (86.1) | 180 (90.0) |
|  | Moderate-intensity infection | 0 (0) | 20 (13.9) | 20 (10.0) |
|  | Heavy-intensity infection | 0 (0) | 0 (0) | 0 (0) |
| Infected with *Ascaris lumbricoides* | | 171 (67.6) | 122 (67.0) | 293 (67.4) |
|  | Light-intensity infection | 72 (42.1) | 60 (49.2) | 132 (45.1) |
|  | Moderate-intensity infection | 93 (54.4) | 61 (50.0) | 154 (52.6) |
|  | Heavy-intensity infection | 6 (3.5) | 1 (0.8) | 7 (2.4) |
| Infected with hookworm | | 11 (4.4) | 5 (2.8) | 16 (3.7) |
| Any STH infection | | 182 (71.9) | 160 (87.9) | 342 (78.6) |
|  | Double infections | 50 (27.5) | 105 (65.6) | 155 (45.3) |
|  | Triple infections | 3 (1.7) | 3 (1.9) | 6 (1.8) |
| Other helminth infections** | | 26 (10.3) | 22 (12.1) | 48 (11.0) |
|  | *Enterobius vermicularis* | 25 (9.9) | 20 (11.0) | 45 (10.3) |
|  | *Hymenolepis nana* | 2 (0.8) | 3 (1.7) | 5 (1.2) |

*Including all children with at least one stool sample analysed; **these infections were not systematically documented, as they are non-target parasites.
